# Supplementary material for: Sport Participation and Academic Performance in Young Elite Athletes
Source: Int J Environ Res Public Health. 2022 Nov 25;19(23):15651. doi: 10.3390/ijerph192315651 (PMC9737165; doi:10.3390/ijerph192315651)
Supplement: Supplementary file 1 [file ijerph-19-15651-s001.zip › ijerph-2012822-supplementary.pdf]

Supplementary Materials

Table S1. Academic performance of elite athletes depending on the sport static and dynamic level from Mitchel et al. classification [32].

	**Low static and Dynamic (n = 48)**	**Low-Moderate Static and Dynamic (n = 61)**	**Moderate static and Dynamic (n = 169)**	**Moderate-High Static and Dynamic (n = 167)**	**High Static and Dynamic (n = 38)**	***p*-Value**
Baccalaureate GPA	7.07 ± 1.1	7.13 ± 1.3	6.80 ± 1.1 [a]	7.09 ± 1.2 [a]	6.89 ± 1.1	0.095
Standardized UEE grade	6.02 ± 1.6 [a,b]	6.86 ± 2.2 [a]	6.38 ± 2.1	6.83 ± 2.3 [b]	6.10 ± 2.3	0.195

Sports classification from Mitchell et al. [32]. Data are shown as mean ± SD. Analyses were adjusted for sex, type of Baccalaureate, UEE call and academic year. Abbreviations: UEE, University Entrance Examinations. [a,b]: significant post-hoc differences between groups.

Mitchell, Blomqvist and Haskell [44] developed a sports classification according to the type and intensity of exercise performed. Exercise was divided into dynamic (isotonic) and static (isometric). This classification was updated in 2005 [32], and it has been widely used by many authors [45,46]. Sports were categorized attending to the updated classification: low static and dynamic (i.e., golf), low-moderate static and dynamic (i.e., archery and fencing), moderate static and dynamic (i.e., karate, badminton), moderate-high static and dynamic (i.e., basketball, swimming) and high static and dynamic (i.e., triathlon, rowing). Academic performance differences are presented according to the sport static and dynamic levels (Table S1).

Table S2. Academic performance of elite athletes in accordance with the body muscle groups involved in different sports.

	**Upper Limbs (n = 123)**	**Lower Limbs (n = 95)**	**Both (n = 265)**	***p*-Value**
Baccalaureate GPA	6.98 ± 1.1	6.99 ± 1.1	6.97 ± 1.2	0.709
Standardized UEE grade	6.28 ± 1.9	6.53 ± 2.2	6.67 ± 2.3	0.456

Data are shown as mean ± SD. Analyses were adjusted for sex, type of Baccalaureate, UEE call and academic year. Abbreviations: UEE, University Entrance Examinations.

With reference to the body muscle groups, some authors have classified the sports considering the involvement of the upper limbs and lower limbs in different sports' movements [36,37]. The academic performance of elite athletes following upper limbs, lower limbs and both is presented in Table S2.

Table S3. Academic performance of elite athletes depending on the sport classification from Bouchard, Brunelle and Goubat [33].

	Individual (*n* = 299)	Team (*n* = 151)	Combat Sport (*n* = 33)	*p*-Value
Baccalaureate GPA	6.95 ± 1.1	7.08 ± 1.2	6.72 ± 1.1	0.656
Standardized UEE grade	6.45 ± 2.2	6.74 ± 2.2	6.43 ± 1.9	0.541

Sports classification based on Bouchard, Brunelle and Goudbout [33]. Data are shown as mean ± SD. Analyses were adjusted for sex, type of Baccalaureate, UEE call and academic year. Abbreviations: UEE, University Entrance Examinations.

Elite athletes' academic performance following the sports classification from Bouchard, Brunelle and Goudbout [33], who classified the sports into individual (e.g., triathlon and horse riding), team (e.g., hockey and baseball) and combat sports (e.g., judo and taekwondo) is shown in Table S3. This classification has been used by many authors, such as Rawat and Bangari [47].

Table S4. Academic performance of elite athletes in accordance with the sport classification from Matveev [34].

	Acyclic Sport (*n* = 101)	Submaximum Endurance (*n* = 65)	Upper- and Mid-Endurance (*n* = 38)	Team Sport with High Intensity and Constant Pauses of Time (*n* = 105)	Team Sport with High Duration and Few Pauses of Time (*n* = 78)	Combat Sport (*n* = 46)	Complex Sport with Multiple Test (*n* = 50)	*p*-Value
Baccalaureate GPA	7.02 ± 1.1	7.08 ± 1.1	7.08 ± 1.2	7.16 ± 1.2 [a,b]	6.79 ± 1.1 [a]	6.87 ± 1.3	6.68 ± 1.0 [b]	0.142
Standardized UEE grade	6.26 ± 1.8 [a,b,c]	6.86 ± 2.2 [d]	7.11 ± 2.6 [a,e]	6.98 ± 2.3 [b,f]	6.38 ± 2.0 [g]	6.68 ± 2.3 [h]	5.43 ± 2.1 [c,d,e,f,g,h]	**0.001**

Sports classification based on Matveev [34]. Data are shown as mean ± SD. Analyses were adjusted for sex, type of Baccalaureate, UEE call and academic year. Abbreviations: UEE, University Entrance Examinations. [a,b,c,d,e,f,g,h]: significant post-hoc differences between groups.

Matveev [34] classified sports into acyclic sports (power and maximum intensity, i.e., weightlifting and sprint in athletics), submaximum endurance (i.e.,100 m in swimming and 800 m in athletics), upper- and mid-endurance (i.e., cross-country in athletics and 1500 m in swimming), team sports with high intensity and constant pauses of time (i.e., basketball and handball), team sports with high duration and few pauses of time (i.e., football), combat sports (i.e., karate and fencing) and complex sports with multiple tests (i.e., triathlon and artistic gymnastics). Table S4 shows the academic performances from elite athletes following Matveev's classification.

Table S5. Academic performance of elite athletes in accordance with the sport classification from Parlebas: environment, partner and adversary [35].

	Alone in Stable Environment (*n* = 113)	Alone in Uncertain Environment (*n* = 97)	With Partner in Stable Environment (*n* = 34)	With Partner in Uncertain Environment (*n* = 4)	With Adversary in Stable Environment (*n* = 92)	With Partner and Adversary in Stable Environment (*n* = 136)	With Partner and Adversary in Uncertain Environment (*n* = 7)	*p*-Value
Baccalaureate GPA	6.98 ± 1.1	7.05 ± 1.1	6.78 ± 1.1	7.05 ± 1.2	6.82 ± 1.2	7.09 ± 1.2	6.76 ± 1.0	0.877
Standardized UEE grade	6.64 ± 2.4	6.08 ± 1.9 [a]	6.12 ± 1.9	6.98 ± 2.7	6.64 ± 2.2	6.77 ± 2.2 [a]	7.41 ± 2.2	0.466

Sports classification based on Parlebas [35]. Data are shown as mean ± SD. Analyses were adjusted for sex, type of Baccalaureate, UEE call and academic year. Abbreviations: UEE, University Entrance Examinations. [a]: significant post-hoc differences between groups.

Parlebas [35] suggested a sports classification based on the environment (stable environment, i.e., tennis and karate vs. uncertain environment, i.e., swimming and skiing), status of a partner (with partner, i.e., hockey and water polo vs. without partner, i.e., athletics and karate) and adversary (with adversary, i.e., judo and baseball and without adversary, i.e., cycling and orienteering). Table S5 presents the classification considering these three variables.
